# SAA1/FPR2 signaling between keratinocytes and neutrophils sustains chronic inflammation in Sweet syndrome

**DOI:** 10.1172/JCI193566

**Published:** 2025-08-19

**Authors:** Jianhe Huang, Satish Sati, Olivia Ahart, Emmanuel Rapp-Reyes, Linda Zhou, Robert G. Micheletti, William D. James, Misha Rosenbach, Thomas H. Leung

**Affiliations:** 1 Department of Dermatology, University of Pennsylvania School of Medicine, Philadelphia, Pennsylvania, USA.; 2 Corporal Michael Crescenz Veterans Affairs Medical Center, Philadelphia, Pennsylvania, USA.

**Keywords:** Dermatology, Immunology, Innate immunity, Neutrophils, Skin

## Abstract

Sweet syndrome (also known as acute febrile neutrophilic dermatosis) is a rare inflammatory skin disorder characterized by erythematous plaques with a dense dermal neutrophilic infiltrate. The first-line therapy remains oral corticosteroids, which suppresses inflammation nonspecifically. Although neutrophils are typically short-lived, how they persist in Sweet syndrome skin and contribute to disease pathogenesis remains unclear. Here, we identify a previously unrecognized population of antigen-presenting cell–like (APC-like) neutrophils expressing MHC class II genes that are uniquely present in Sweet syndrome skin but absent in healthy tissue and the circulation. Keratinocytes extended neutrophil lifespan 10-fold in coculture experiments and drove the emergence of an APC-like phenotype in approximately 30% of neutrophils, mirroring observations in patients’ lesions. Mechanistically, keratinocyte-derived serum amyloid A1 (SAA1) signals through the formyl peptide receptor 2 (FPR2) on neutrophils to promote their survival. These long-lived neutrophils actively orchestrate local immune responses by recruiting T cells and inducing cytokine production. Strikingly, dual blockade of SAA1/FPR2 signaling restores neutrophil turnover to baseline levels, with efficacy comparable to high-dose corticosteroids. These findings uncover a keratinocyte/neutrophil/T cell axis that sustains chronic inflammation in Sweet syndrome and highlight the SAA1/FPR2 pathway as a promising target for precision therapy.

## Abstract 2

In Sweet syndrome, skin cells keep immune cells alive longer, worsening inflammation. Blocking a newly discovered pathway may offer a safer, targeted treatment to steroids.

## Graphical abstract

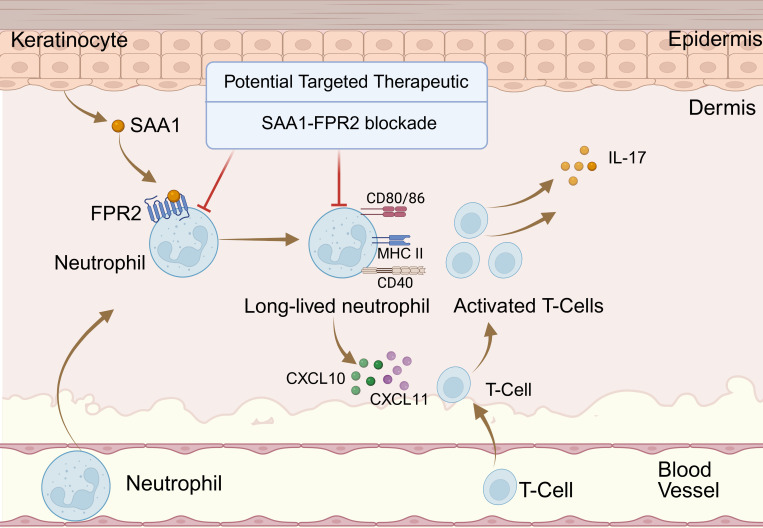

## Introduction

Sweet syndrome (also referred to as acute febrile neutrophilic dermatosis) presents with fever, leukocytosis, and a painful raised red rash (1, 2). Systemic multiorgan involvement may occur in severe cases, which carries a considerable mortality risk. Histologic evaluation of the skin lesions reveals a dense dermal neutrophilic infiltrate. We previously showed that a neutrophil-specific mutation in the PIK3R1/AKT signaling pathway renders them more sensitive to IL-1β and drives increased recruitment to the skin (3). However, how and why neutrophils are persistently active in Sweet syndrome, and the role they play in the proinflammatory milieu, remains unclear (4, 5).

Systemic corticosteroids are effective and remain the first-line therapy for treatment. However, their chronic use results in well-characterized serious side effects, including diabetes, weight gain, and spontaneous vertebral fractures. Steroid-sparing antiinflammatory agents such as colchicine, dapsone, potassium iodide, and spesolimab may also be used for management, but their efficacy remains limited (4, 6). No targeted therapeutics exist that are safe and effective for Sweet syndrome.

Neutrophils are considered to be terminally differentiated, short-lived cells that survive only a few hours to days in the circulation, primarily functioning as innate immune effectors that nonspecifically eliminate pathogens (7). However, emerging evidence suggests that neutrophils may act in a more discriminating fashion, with distinct subpopulations exhibiting specialized immune roles (reviewed in ref. 8). During homeostasis, circulating human neutrophils do not express cell-surface markers related to antigen presentation, including HLA complex II or other costimulatory molecules, such as CD40, CD80, or CD86. However, these marker genes may be induced in vitro when neutrophils are stimulated with inflammatory mediators. Tissue-infiltrating human neutrophils were also found ex vivo to express MHC marker genes in inflamed joint fluid (9, 10). Human antigen-presenting cell–like (APC-like) neutrophils (APC-neutrophils) were shown in vitro to activate memory T cell proliferation and cytokine production, although ex vivo studies remain unclear (11, 12). In contrast, APC-neutrophils have been better characterized in mice. Mouse APC-like neutrophils were shown to process protein antigen, load it onto HLA molecules, and activate CD4^+^ and CD8^+^ T cells (13–15). Despite these advances, a formal role for human APC-neutrophils driving disease pathogenesis remains unclear.

We performed high-resolution analysis of skin and blood samples from patients with Sweet syndrome to unbiasedly assess immune-mediated disease mechanisms. We show that keratinocytes increased neutrophil longevity, with approximately 30% of these long-lived neutrophils adopting an APC-like phenotype. These long-lived neutrophils recruited and activated T cells to establish a self-perpetuating axis that sustained chronic inflammation.

## Results

### Immune cell landscape in Sweet syndrome.

**Figure 1:**
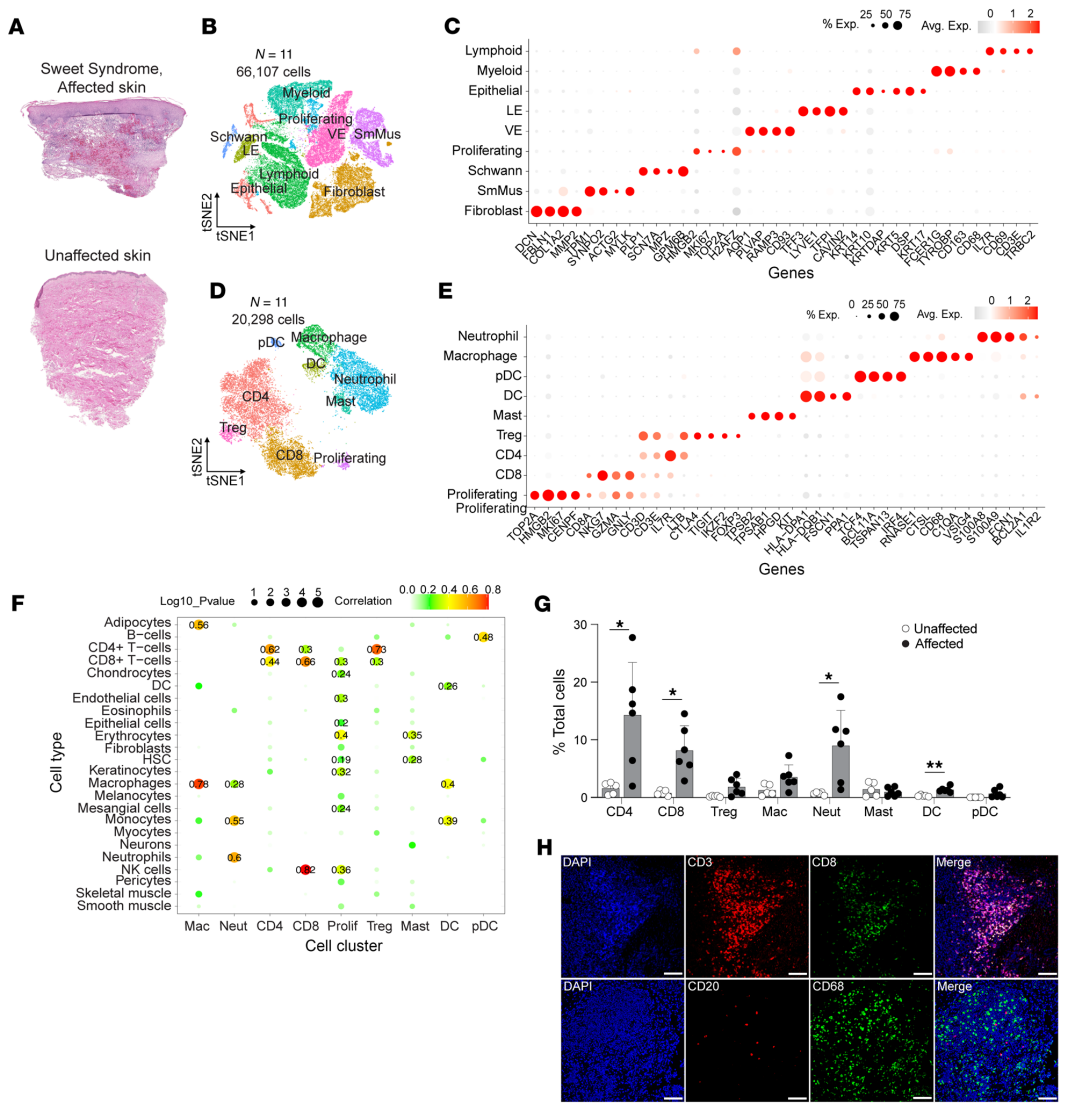
Immune cell landscape in Sweet syndrome. (**A**) H&E staining of affected and unaffected skin. (**B**) *t*-SNE plot depicting all cell clusters (*n* = 6 for affected and *n* = 5 for unaffected skin). (**C**) Marker genes defining cell types. Dot size reflects the percentage of cells expressing the gene, and color illustrates the level of gene expression. (**D**) *t*-SNE plot depicting immune cell subclusters. (**E**) Marker genes defining immune cell types. Dot size reflects the percentage of cells expressing the gene, and color illustrates the level of gene expression. (**F**) Decoding cell type specificity correlation plot for cell type enrichment analysis of different cell clusters. The *y*-axis shows the main cell types identified from the BlueprintEncode database. The color scale represents the Pearson correlation coefficient, and dot sizes represent the –log_10_-transformed *P* value. Prolif, proliferating. (**G**) Bar graph shows relative contribution of immune cell subtype as the percentage of total cells. Two-tailed, unpaired Student’s *t* test. *P < 0.05 and **P < 0.01 (**H**) Representative immunofluorescence staining of 4 diseased and 3 normal skin samples confirming populations of macrophages (CD68^+^), B cells (CD20^+^), and T cells : (CD3^+^, CD8^+^) in Sweet syndrome dermis. Scale bars: 100 μm. Avg. Exp., average expression; HSC, hematopoietic stem cells; pDC, plasmacytoid dendritic cells; Neut, neutrophil; LE, lymphatic endothelial; Mac, macrophage; SmMus, smooth muscle; VE, vascular endothelial.

We collected 4 mm skin biopsies (affected and unaffected skin) and blood from 6 patients with Sweet syndrome with clinically active and histologically validated skin disease, who were new to our service (Figure 1A, Supplemental Figure 1A, and Supplemental Table 1; supplemental material available online with this article; https://doi.org/10.1172/JCI193566DS1). Our cohort was composed of 5 males and 1 female, with a mean age of 67 years. Five patients had idiopathic Sweet syndrome, and 1 patient had malignancy-associated Sweet syndrome. Unaffected skin biopsies from the same donor served as control samples. To ensure robust neutrophil capture, we optimized our tissue dissociation protocol to achieve greater than 40% neutrophil viability in the control experiments (Supplemental Figure 1B). We generated 66,107 single-cell RNA-Seq (scRNA-Seq) profiles (Supplemental Table 2). Unsupervised clustering of scRNA-Seq profiles identified 9 cell types based on marker gene identification, lineage marker genes, and mapping to single-cell databases (Figure 1, B and C, and Supplemental Figure 1, C–E). The identified cell types were shared among affected and unaffected samples (Supplemental Figure 1F and Supplemental Tables 2 and 3).

To track the immune cell contributions, we subclustered the immune cell populations and identified 9 different immune cell subtypes (Figure 1, D and E, Supplemental Figure 2, A and B, and Supplemental Tables 2 and 3). We confirmed these cell types by mapping their gene expression profiles to Blueprint ENCODE and Monaco Immune single-cell databases (Figure 1F and Supplemental Figure 2C). As expected, affected skin from patients with Sweet syndrome contained significantly more neutrophils than did unaffected skin (Figure 1G, Supplemental Figure 2D, and Supplemental Table 2). Surprisingly, we saw significantly more recruitment of T cells and DCs, although the number of DCs was very low overall. Immunofluorescence staining confirmed the recruitment of T cells (CD3^+^ or CD8^+^) and macrophages (CD68^+^) in affected Sweet syndrome skin compared with healthy control skin (Figure 1H and Supplemental Figure 2E). Taken together, this unbiased approach revealed that neutrophils and T cells were the 2 dominant immune cell populations that were recruited in the affected Sweet syndrome skin.

### Neutrophils in Sweet syndrome skin adopt an APC-like state.

**Figure 2:**
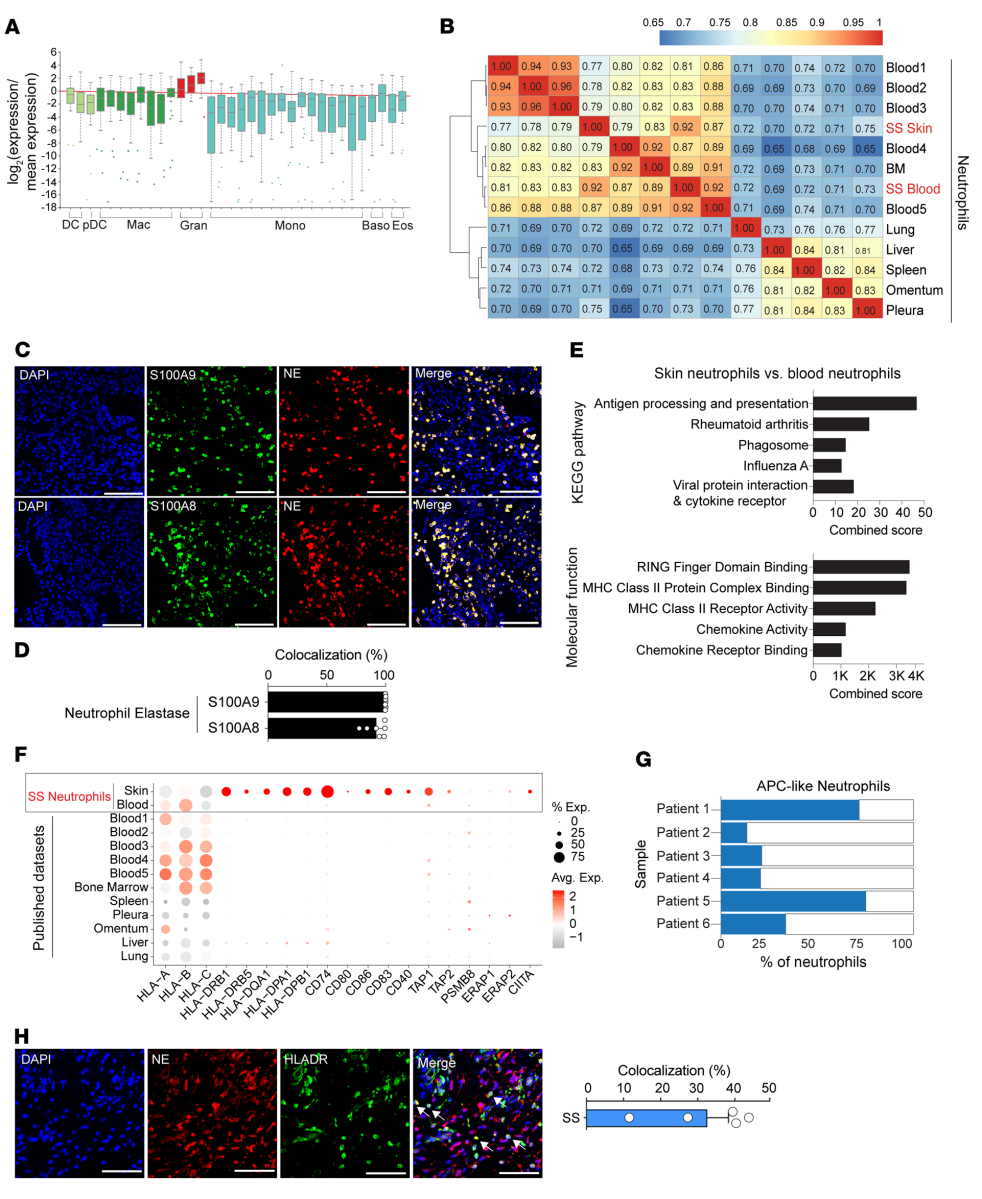
APC-like neutrophils are specifically recruited to Sweet syndrome–affected skin. (**A**) Gene expression of neutrophil single-cell cluster matched the granulocyte definition in the Immunological Genome Project database. Baso, basophil, Eos, eosinophil; Gran, granulocyte; Mono, monocyte. (**B**) Spearman correlation heatmap validating neutrophil identity by comparing Sweet syndrome (SS) neutrophils against published human neutrophil datasets from healthy individuals. Sweet syndrome skin, Sweet syndrome skin neutrophils; Sweet syndrome blood, Sweet syndrome blood neutrophils, BM, and other tissue sources as indicated. Color scale represents the correlation coefficient. (**C**) Immunofluorescence staining images of Sweet syndrome skin neutrophils demonstrating coexpression of neutrophil-specific markers (S100A8, S100A9) and NE. (**D**) Quantification of immunofluorescence staining. (**E**) Gene ontogeny analysis of Sweet syndrome skin neutrophils versus blood neutrophils. (**F**) Dot plot comparing the expression of antigen-presenting genes in Sweet syndrome skin neutrophils, Sweet syndrome blood neutrophils, and published datasets. Dot size reflects the percentage of cells expressing the gene, and the color illustrates the level of gene expression. (**G**) Percentage of neutrophils coexpressing the top-3 MHC II–related genes in individual patient samples. (**H**) Representative images and quantification of immunofluorescence staining from 5 Sweet syndrome skin samples confirming coexpression of MHC II and NE. Scale bars: 100 μm (**C** and **H**).

scRNA-Seq analysis of neutrophils has been challenging because of their fragility, short lifespan, and low RNA content, and most published single-cell atlases do not include them. To confirm the identity of our neutrophil population, we used 4 complementary approaches. (a) We overlaid the gene expression profiles from our identified neutrophil subcluster with the mouse Immunological Genome Project (ImmGen) database, which revealed a strong match with the ImmGen granulocyte (neutrophil) profile (Figure 2A). (b) We validated our neutrophils against multiple published human neutrophil datasets from healthy individuals (16–19). Using Spearman correlation analysis, our Sweet syndrome blood and skin neutrophils showed a strong correlation (*r* > 0.8) with published sorted healthy neutrophil transcriptomes from multiple independent studies, as well as a strong correlation (*r* > 0.7) with neutrophils isolated from different tissues (Figure 2B). (c) We compared our gene expression profiles against the Human Cell Atlas (HCA) of healthy neutrophils purified from different tissues. Neutrophil marker genes identified in the HCA were highly enriched in our Sweet syndrome neutrophil cluster, including the HCA’s top 15 neutrophil-specific marker genes, e.g., *S100A8*, *S100A9*, *CSF3R*, *FPR1*, and *GCA*. (Supplemental Figure 3, A and B) (16, 17). (d) We performed immunofluorescence staining for 2 of these marker genes, *S100A8* and *S100A9*. These markers showed strong colocalization with neutrophil elastase (NE) (Figure 2, C and D) and myeloperoxidase (MPO) (Supplemental Figure 4, A and B). Taken together, we correctly identified a neutrophil subpopulation within our scRNA-Seq analysis.

We also collected blood from our 6 patients with Sweet syndrome, purified their neutrophils by magnetic bead sorting, and created an 18,721 single-cell RNA-Seq dataset (Supplemental Figure 4, C and D, and Supplemental Table 3). We annotated these cells using standard methods. The human PBMC single-cell reference atlas created by CITE-Seq does not contain neutrophil data (18). Our blood neutrophils also exhibited a strong correlation against published datasets (Figure 2B), and the top 15 marker genes identified in our skin neutrophil analysis were also well represented in our blood neutrophil dataset (Supplemental Figure 4E).

Next, we compared Sweet syndrome skin neutrophils against blood neutrophils. Gene ontogeny pathways and transcriptome analysis revealed that skin neutrophils had enrichment for antigen processing and presentation as well as MHC class II protein complex binding and activity (Figure 2E and Supplemental Tables 4 and 5). Compared with Sweet syndrome blood neutrophils, skin-infiltrating neutrophils induced multiple MHC-related genes in a robust fashion, including *HLA-DRB1*, *HLPA-DPA1*, *HLA-DPB1*, *CD74*, *CD83*, *TAP1*, and *TAP2* (Figure 2F and Supplemental Figure 4F). We also compared our dataset with 4 publicly available datasets for purified human healthy neutrophils from blood, bone marrow, and other tissues (16, 17, 19). Despite sharing many neutrophil marker genes, none of the neutrophils in these datasets expressed marker genes related to MHC II antigen presentation (Figure 2F, lower portion, left side). To ensure that our tissue-processing protocol did not artificially induce this phenotype, we treated healthy blood neutrophils with our tissue dissociation protocol and found no upregulation of MHC II genes by quantitative PCR (qPCR) (Supplemental Figure 4G). We demonstrated that between approximately 20% and 75% of skin neutrophils from individual Sweet syndrome patients coexpressed the top 3 MHC II genes (*HLA-DRB1*, *HLA-DPA1*, *HLA-DPB1*), with an overall average of approximately 40% of neutrophils (Figure 2G). Finally, immunofluorescence staining of Sweet syndrome skin confirmed colocalization of MHC II and NE protein expression, with approximately 30% of neutrophils expressing both marker genes (Figure 2H). Taken together, we conclude that approximately 30% of skin-infiltrating neutrophils in Sweet syndrome adopt an APC-like phenotype.

### Keratinocyte coculture induces neutrophil longevity and MHC II gene expression.

**Figure 3:**
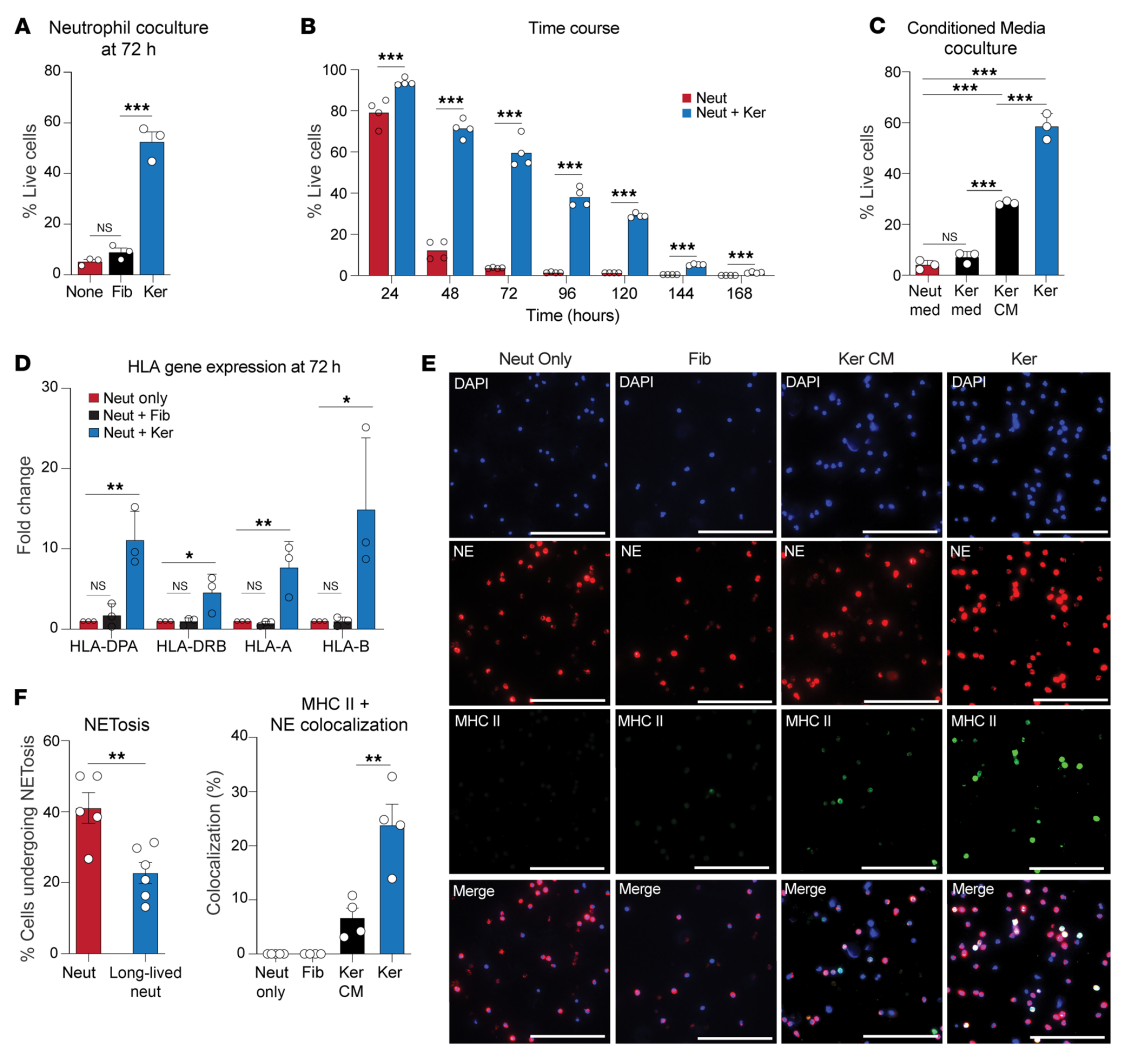
Keratinocytes induce healthy neutrophils to adopt an APC-like state. (**A**) Coculture of healthy neutrophils with keratinocytes (Ker), and not fibroblasts (Fib), prolonged neutrophil lifespan. (**B**) Time course of neutrophil lifespan for healthy and cocultured neutrophils. (**C**) Keratinocyte CM prolonged neutrophil survival at 72 hours. (**D**) HLA genes were induced in neutrophils cocultured with keratinocytes. (**E**) Representative immunofluorescence staining and quantification confirming the expression of MHC II protein in neutrophils exposed to keratinocytes or keratinocyte CM. The experiment was repeated 4 times. Scale bars: 100 μm. (**F**) Decreased NETosis in long-lived neutrophils compared with healthy neutrophils (*n* = 5 in neutrophils; *n* = 6 in long-lived neutrophils). Two-tailed, unpaired Student’s *t* test (**A**, **B**, and **F**) and 1-way ANOVA with individual comparisons (**C** and **D**). *P < 0.05, ** *P* < 0.01, and ***P < 0.001.

We were intrigued that neutrophils adopted this activation state only after entry into the skin. To elucidate the molecular mechanisms driving this behavior, we established an in vitro cell culture system with primary human neutrophils and measured cell viability. As expected, primary human neutrophils exhibited a limited lifespan, with the majority undergoing cell death by 48 hours and only 5% remaining at 72 hours (Figure 3, A and B). To simulate entry into the skin, we cocultured neutrophils with primary healthy human keratinocytes or fibroblasts. When exposed to keratinocytes, the healthy neutrophil lifespan increased 10-fold, with more than 50% remaining after 72 hours and 30% remaining after 120 hours (Figure 3, A and B). This effect was specific to keratinocytes, because neutrophils cocultured with fibroblasts did not exhibit any change in lifespan. We next performed a conditioned media (CM) experiment to assess whether physical contact between the cell types was necessary. Keratinocyte CM increased neutrophil lifespan 5-fold compared with control media, with 25% of neutrophils alive after 72 hours (Figure 3C). Thus, a keratinocyte-secreted factor or factors increased the lifespan of cultured neutrophils.

Next, we assessed whether this increased lifespan may be related to an APC-like activation state. Indeed, neutrophils cocultured with keratinocytes or keratinocyte CM expressed higher levels of MHC-related transcripts (*HLA-DPA*, *HLA-DRB*, *HLA-A*, *HLA-B*) and MHC II protein expression (Figure 3, D and E). Similar to infiltrating neutrophils in human skin, approximately 25% of these long-lived neutrophils expressed MHC II protein, and this percentage remained similar over time (Figure 3E and Supplemental Figure 4H). Neutrophil extracellular traps (NETs) are typically secreted by neutrophils to kill extracellular pathogens. We found that long-lived neutrophils displayed reduced spontaneous NETosis compared with control neutrophils, confirming a distinct functional adaptation (Figure 3F and Supplemental Figure 5A). Taken together, neutrophils exposed to keratinocytes adopted a long-lived activation state, and a subset of these cells adopt an APC-like phenotype.

### Long-lived neutrophils orchestrate T cell recruitment and activation.

**Figure 4:**
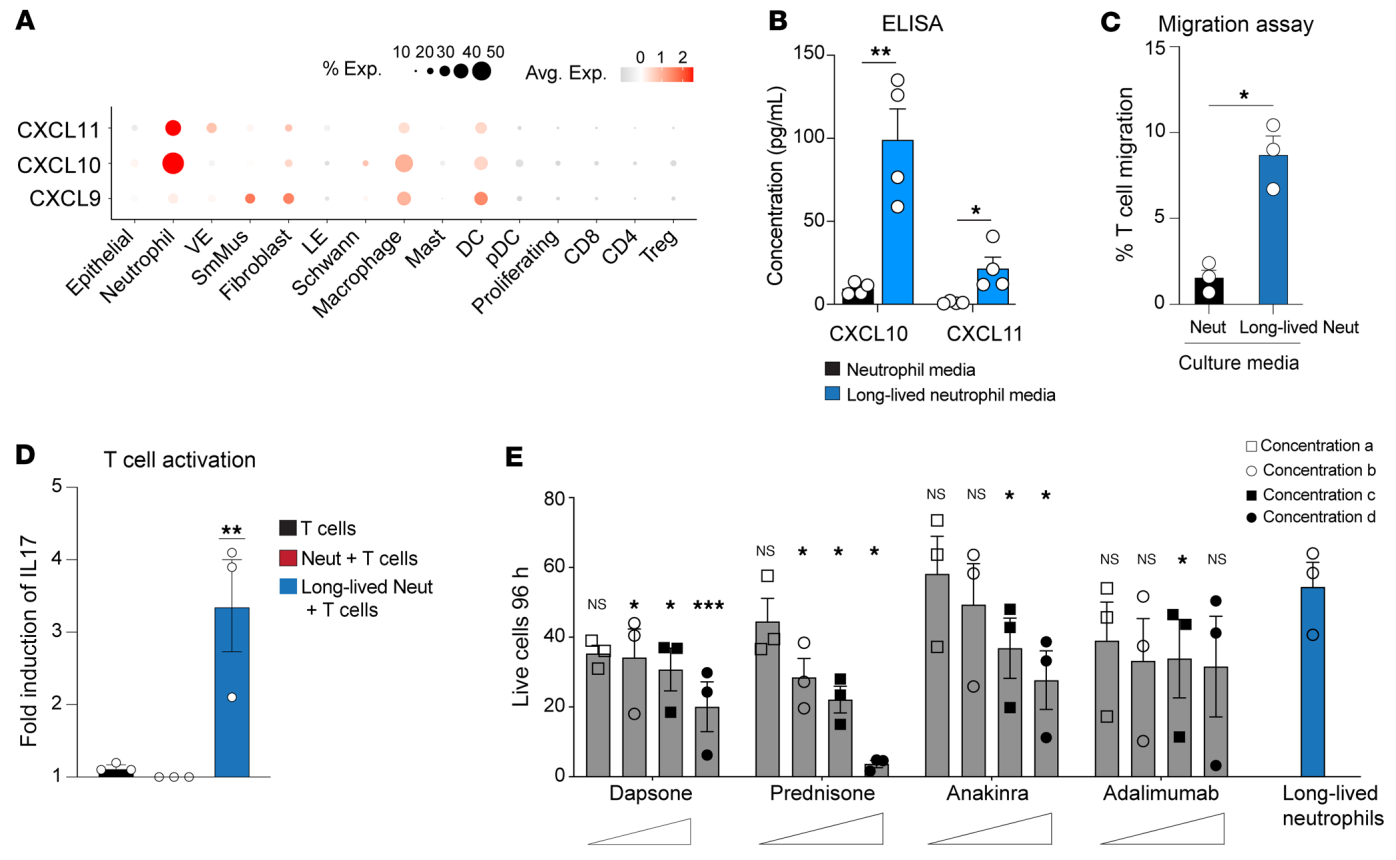
Long-lived neutrophils orchestrate T cell recruitment and activation. (**A**) Dot plot showing the expression of T cell chemoattractants (*CXCL9*, *CXCL10*, *CXCL11*) across different cell types in Sweet syndrome skin. Dot size reflects the percentage of cells expressing the gene, and the color illustrates the level of gene expression. (**B**) ELISA quantification of CXCL10 and CXCL11 secretion from neutrophil-keratinocyte coculture (long-lived neutrophils) versus neutrophils alone. *n* = 4 independent experiments. Data are shown as the mean ± SEM. (**C**) T cell migration assay comparing the response to CM from neutrophil-keratinocyte cocultures or neutrophils alone. Data are represented as a percentage of migrated T cells (mean ± SEM) from 3 independent donors. (**D**) Long-lived neutrophils induced higher *IL17* transcript levels in T cells. *n* = 3. (**E**) Sweet syndrome therapeutics reduce APC-neutrophil lifespan in a dose-dependent manner. *n* = 3 for each condition. Data are shown as the mean ± SEM. *P < 0.05, **P < 0.01, and ***P < 0.001, by 2-tailed, unpaired Student’s *t* test comparing control-treated cells (**B**, **C**, and **E**) and 1-way ANOVA (**D**).

Our scRNA-Seq analysis revealed that neutrophils, but not epithelial cells, in Sweet syndrome skin expressed elevated levels of CXCL10 and CXCL11, which are well-established T cell chemoattractants (Figure 4A). We wanted to assess whether long-lived neutrophils also secreted more CXCL10 and CXCL11. ELISA of cultured media demonstrated that long-lived neutrophil cultures contained higher levels of CXCL10 and CXCL11 than did healthy neutrophils alone (Figure 4B). Moreover, this cultured media from long-lived neutrophils enhanced T cell migration in a Transwell assay compared with media from healthy neutrophils alone (Figure 4C). Thus, long-lived neutrophils enhanced T cell recruitment through secretion of CXCL10 and CXCL11.

Multiple studies have demonstrated that human APC-neutrophils may stimulate cytokine production from CD4^+^ and CD8^+^ T cells (reviewed in ref. 8). To test whether keratinocyte-induced, long-lived neutrophils may also activate T cells, we cocultured human long-lived neutrophils or primary neutrophils with autologous T cells. Compared with primary neutrophils, long-lived neutrophils robustly induced T cells to express more *IL17* transcripts (Figure 4D). Taken together, long-lived neutrophils recruited and stimulated cytokine production from T cells.

We next wanted to test whether established Sweet syndrome therapeutics shorten the lifespan of long-lived or healthy neutrophils. Prednisone and anakinra (IL-1 receptor [IL-1R] antagonist) reduced long-lived neutrophil lifespan in a dose-dependent manner, with only the highest dose of prednisone restoring neutrophil turnover to baseline levels (Figure 4E). In contrast, all steroid-sparing agents, including adalimumab (TNF-α inhibitor), dapsone, and anakinra were markedly less effective in reducing healthy or long-lived neutrophil survival (Figure 4E and Supplemental Figure 5B).

### Keratinocyte-secreted SAA1 promotes neutrophil survival through FPR2 signaling.

**Figure 5:**
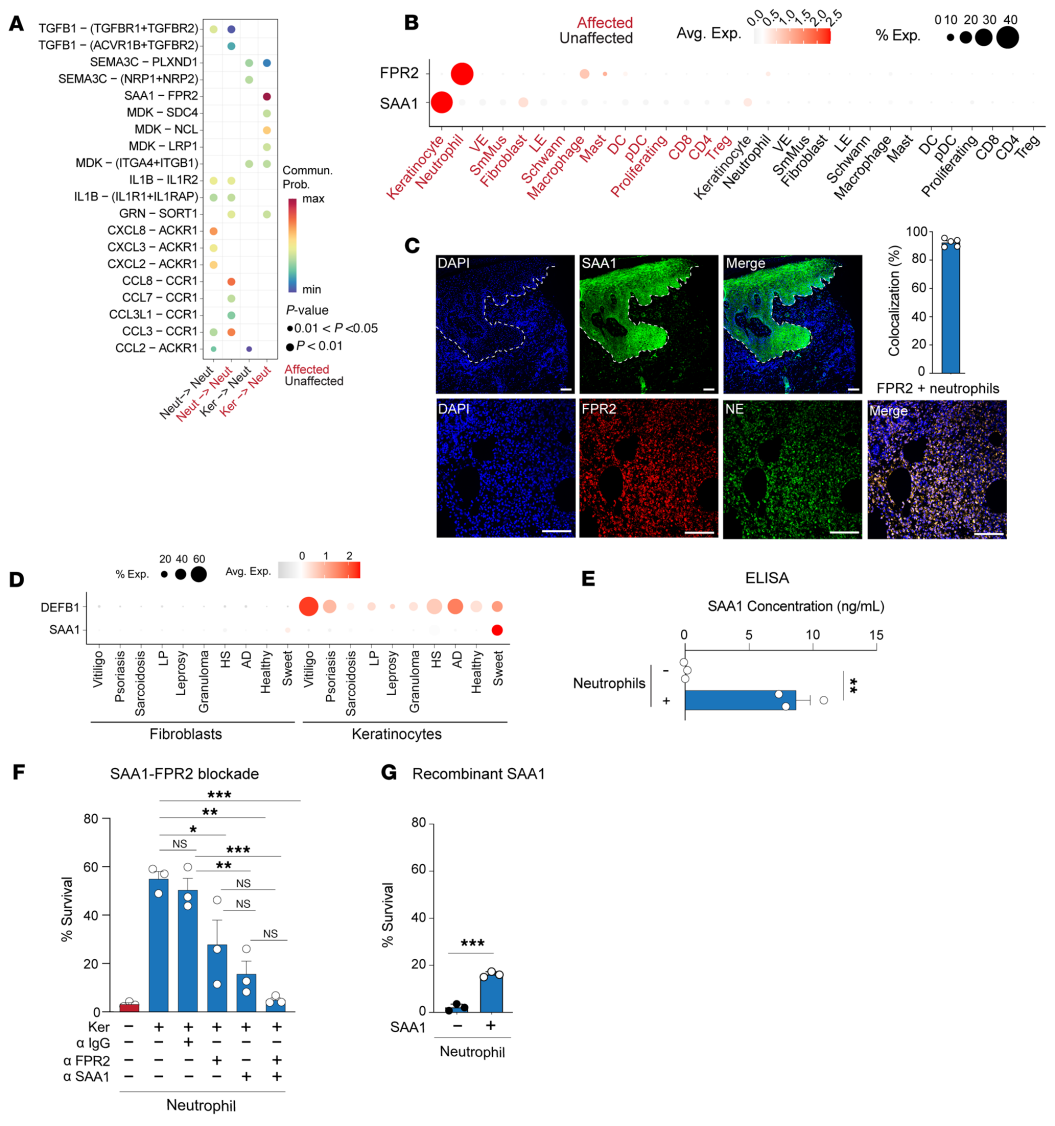
SAA1/FPR2 signaling is necessary and sufficient for neutrophils to adopt a long-lived state. (**A**) Ligand-receptor analysis reveals keratinocyte- and neutrophil-specific interactions. Keratinocytes expressed *SAA1* transcripts and neutrophils expressed the *FPR2* receptor (red dot, right-most column). (**B**) Dot plot demonstrating predominantly cell-specific expression of *SAA1* and *FPR2* transcripts. The dot size reflects the percentage of cells expressing the gene, and the color illustrates the level of gene expression. (**C**) Representative immunofluorescence staining images and quantification from 5 diseased and 5 control samples, confirming the expression of SAA1 and FPR2 in keratinocytes and neutrophils, respectively. Scale bars: 100 μm. (**D**) Dot plot comparing keratinocyte *SAA1* and the control gene *DEFB1* in different inflammatory skin conditions. The dot size reflects the percentage of cells expressing the gene, and the color illustrates the level of gene expression. (**E**) SAA1 secretion measured by ELISA in healthy neutrophils or healthy neutrophils exposed to keratinocytes. (**F**) Antibodies blocking SAA1 and FPR2 restored long-lived neutrophil lifespan to WT neutrophil levels. (**G**) Recombinant human SAA1 increased neutrophil survival at 72 hours (*n* = 3 independent donors). Data indicate the mean ± SEM. *P < 0.05, **P < 0.01, and ***P < 0.001, by 2-tailed, unpaired Student’s *t* test (**E** and **G**) and 1-way ANOVA with individual comparisons (**F**).

To identify the keratinocyte-secreted factor responsible for the increased neutrophil lifespan, we returned to our scRNA-Seq dataset and focused on interactions between keratinocytes and neutrophils. Cell-specific ligand-receptor analysis identified serum amyloid A1 (SAA1) binding to formyl peptide receptor 2 (FPR2) as the strongest interaction from keratinocytes to neutrophils (Figure 5A, red dot). SAA1 is a well-established, major acute-phase protein produced in response to infection, injury, and malignancy. We confirmed that SAA1 and FPR2 transcripts and proteins are predominately expressed by keratinocytes and neutrophils in human Sweet syndrome skin, respectively (Figure 5, B and C, and Supplemental Figure 5C). Notably, when we compared *SAA1* expression in keratinocytes across different inflammatory skin conditions, Sweet syndrome keratinocytes exhibited a unique *SAA1* induction profile compared with another established acute-phase gene, defensin β 1 (*DEFB1*) (Figure 5D). Thus, SAA1 production by keratinocytes may be relevant to Sweet syndrome pathogenesis.

To determine whether SAA1/FPR2 signaling regulates neutrophil survival, we first confirmed that keratinocytes cocultured with neutrophils secrete higher levels of SAA1 protein than keratinocytes cultured alone (Figure 5E). We then treated long-lived neutrophils with neutralizing antibodies against SAA1 or FPR2, or with an IgG control. Inhibition of either SAA1 or FPR2 reduced long-lived neutrophil lifespan by 25%–50%, whereas combined blockade of both molecules reduced neutrophil lifespan to baseline levels, which was comparable to the maximal effect observed with high-dose prednisone (Figure 5F). To test whether SAA1 is sufficient to promote neutrophil survival, we exposed healthy neutrophils to recombinant human SAA1. This treatment extended neutrophil lifespan 4-fold at 72 hours relative to that of untreated controls (Figure 5G). However, recombinant SAA1 alone did not induce the expression of MHC class II genes (*HLA-DRB* and *HLA-DPA*) within 24 hours (Supplemental Figure 5D), suggesting that additional keratinocyte-derived factors were required to drive the full APC-like phenotype. Together, these findings demonstrate that SAA1/FPR2 signaling was necessary and sufficient to promote neutrophil survival but was not sufficient on its own to induce APC-like differentiation. Targeting the SAA1/FPR2 pathway may offer a novel therapeutic strategy for Sweet syndrome.

## Discussion

Our data support a model in which APC-neutrophils perpetuate and sustain inflammation in Sweet syndrome through a self-reinforcing inflammatory loop. First, their extended lifespan enables persistence in the skin, sustaining chronic inflammation, and explains the hallmark-dense dermal neutrophilic infiltrate observed histologically. Second, long-lived neutrophils secrete the T cell chemoattractants CXCL10 and CXCL11, promoting T cell recruitment. This is consistent with our observation of increased T cell infiltration in Sweet syndrome lesions compared with unaffected skin. Although often overshadowed by the dense neutrophilic infiltrate, lymphocytes are consistently observed in the upper dermis, near areas of elevated SAA1 expression (20). Third, long-lived neutrophils induce T cells to produce proinflammatory cytokines such as IL-17, creating a positive feedback loop that sustains tissue inflammation. Consistently, prior work and our scRNA-Seq dataset demonstrated increased expression of IL-17 in human Sweet syndrome skin (20, 21). This reprogramming into long-lived neutrophils resembles the tissue-specific adaptations seen in other immune cell types. We further show that neutrophil infiltration stimulated keratinocyte-derived SAA production, which in turn drove neutrophil reprogramming into long-lived cells. Importantly, both high-dose corticosteroids and pharmacologic blockade of SAA1/FPR2 signaling disrupted this pathogenic loop and restored long-lived neutrophil lifespan to baseline levels. Together, these findings provide a mechanistic framework in which reprogrammed neutrophils sustain chronic cutaneous inflammation in Sweet syndrome.

SAA1 is a well-established acute-phase reactant capable of increasing up to 1,000-fold in the serum during inflammation, infection, or malignancy and is known to modulate cytokine production in diverse contexts (21). However, its role in regulating neutrophil function remains incompletely understood. Prior work showed that breast cancer–derived SAA1 can drive neutrophils to produce immunosuppressive mediators such as IL-10, arginase, and NOS (22). Importantly, we demonstrate that SAA1/FPR2 signaling was necessary for increased neutrophil survival. While SAA1 alone was sufficient to extend neutrophil lifespan, SAA1 was not detectable by ELISA in our keratinocyte CM. Thus, additional keratinocyte-derived factor(s) may also promote neutrophil longevity. Because only dual blockade of SAA1 and FPR2 fully reduced neutrophil lifespan to baseline, these other factors may converge on the FPR2 receptor. Future studies are needed to identify these cofactors and define the molecular mechanisms driving the observed phenotypic shift.

Although keratinocytes induced neutrophils to adopt a long-lived activation state, only a subset of neutrophils expressed APC-related genes. This proportion remained stable at approximately 30% over time, indicating that long-lived and APC-like states represent distinct but related activation programs. We speculate that the APC subset is responsible for recruiting and activating cytokine production from T cells, including IL-17. Prior literature established that APC-like neutrophils may activate T cells through both MHC-dependent and MHC-independent mechanisms. Our experimental designs suggest that APC-neutrophil–mediated T cell recruitment and activation go through an MHC-independent mechanism. However, we cannot formally rule out an MHC-dependent mechanism, and future studies are needed using antigen-pulsed APC-neutrophils with antigen-specific T cells to demonstrate classical MHC-restricted antigen presentation. Because neutrophils typically infiltrate inflamed tissues earlier than do macrophages or DCs, these APC-like neutrophils may play a critical role in initiating and amplifying the immune response. Further work is needed to dissect the regulatory programs that distinguish long-lived neutrophils from their APC-like counterparts.

Our findings are consistent with prior reports that neutrophils in inflamed tissues or lymphoid organs adopt markedly different phenotypes compared with those in circulation. Notably, we did not detect APC-like neutrophils in Sweet syndrome blood, highlighting the importance of local tissue signals in driving this reprogramming. This underscores the need for future studies to prioritize primary tissue-resident cells over circulating cell populations. Our patient cohort included both idiopathic and malignancy-associated Sweet syndrome cases, suggesting that the observed mechanism may be broadly applicable across disease subtypes.

We recognize that this SAA/FPR2 signaling pathway may not be unique to Sweet syndrome. Indeed, coculturing with healthy keratinocytes can induce a long-lived neutrophil phenotype even in the absence of disease. However, we did not observe strong keratinocyte-driven SAA1 expression in other inflammatory skin conditions, including those with known prominent neutrophilic involvement, like psoriasis and hidradenitis suppurativa. One possibility is that in other disorders, neutrophils are primed or activated before entering the skin or are influenced by other cell types or pathogens. Further comparative studies are warranted to determine whether similar mechanisms operate in related and more rare neutrophil-rich dermatoses, including pyoderma gangrenosum.

A longstanding challenge in neutrophil research has been the cell’s short lifespan and fragility, which limits both functional assays and inclusion in single-cell studies. Sweet syndrome, characterized by massive neutrophil infiltration into the skin, provided a unique opportunity to overcome this barrier. We also optimized tissue dissociation protocols to enrich for viable neutrophils, although we acknowledge that cell loss likely occurred and that the absolute number of neutrophils in our single-cell dataset was likely underestimated. We also recognized the necessity for rigorous validation of neutrophils within single-cell datasets to ensure accuracy and reproducibility. Finally, we showed that keratinocyte coculture increased neutrophil lifespan by 10-fold, which could substantially enhance neutrophil-focused research. Future work should explore whether lifespan can be extended further by modifying keratinocyte stimulation or identifying additional FPR2 agonists.

Corticosteroids remain the mainstay treatment for Sweet syndrome, and our findings suggest that part of their efficacy may stem from reducing the survival of pathogenic, long-lived neutrophils. However, some patients develop chronic Sweet syndrome and require long-term steroids, which carry serious adverse side effects. Existing steroid-sparing agents were markedly less effective at reversing neutrophil survival. Importantly, dual blockade of SAA1 and FPR2 restored neutrophil lifespan to baseline levels, comparable to high-dose corticosteroids. These data identify the SAA1/FPR2 axis as a compelling therapeutic target and support further evaluation of this pathway in clinical studies. Finally, our single-cell dataset offers a valuable resource for the research community to further investigate neutrophil biology and skin inflammation.

## Methods

### Sex as a biological variable

We attempted to recruit equal numbers of male and female patients, and these numbers are indicated in the main text when appropriate.

### Experimental model and participant details

#### Study participants (human).

Sweet syndrome diagnosis was confirmed by a team of dermatopathologists and clinicians based on histological assessment, patient history, and clinical phenotype. The patient data and associated demographics are provided in Supplemental Table 1. Demographic information was provided by the participants, and written informed consent was obtained by the investigators. Punch biopsies (4–6 mm) were taken from affected skin and unaffected skin. All biopsies were placed on saline-soaked gauze prior to processing. Whole-blood samples were collected in collection tubes with EDTA (BD). Healthy volunteer neutrophils and T cells were obtained from the Human Immunology Core (HIC) at the University of Pennsylvania.

#### Human tissue processing, single-cell RNA-Seq library preparation and sequencing.

scRNA-Seq was performed as described previously (22, 23). Briefly, the skin-punch biopsies were washed in ice-cold PBS and minced into pieces smaller than 1 mm^3^ using a scalpel in 500 µL serum-free RPMI 1640 media supplemented with DNase I (0.2 mg/mL, 12633012, Thermo Fisher Scientific), 20 mM HEPES, and 0.25 mg/mL Liberase (5401119001, Roche). The suspension was incubated for 60 minutes at 37°C with gentle agitation every 10 minutes. Digestion was terminated by adding 100 μL FBS and 3 μL of 0.5 M EDTA. The cell suspension was filtered through a prewetted 70 μm cell strainer (22-363-548, Thermo Fisher Scientific), and cells were pelleted at 300*g* for 5 minutes at 4°C. Cells were washed twice with ice-cold PBS containing 1% BSA, resuspended in PBS containing 0.04% BSA, and kept on ice until processing.

To minimize neutrophil damage during tissue processing, we systematically tested multiple enzymatic conditions and incubation durations for tissue dissociation to identify optimal parameters that balanced efficiency with cell viability, particularly for neutrophils. We performed control experiments in which purified blood neutrophils from healthy donors were subjected to identical enzymatic treatment conditions. Cell viability was assessed using trypan blue exclusion and flow cytometry with LIVE/DEAD staining. These experiments confirmed that approximately 40% of neutrophils remained viable after enzymatic treatment (Supplemental Figure 1B), demonstrating that a substantial neutrophil population survived our processing protocol intact.

scRNA-Seq was performed using the 10X Chromium 3, version 3.1 kit (1000268, 10X Genomics). The sequencing libraries were prepared per the manufacturer’s protocol and sequenced 2 × 100 bp paired-end run on the Illumina HiSeq 2000/HiSeq 2500 platforms at the BGI America. The raw and processed sequencing data details are given in Supplemental Table 2.

#### Neutrophil isolation.

Neutrophils for all in vitro experiments were isolated from healthy adult volunteers (ages 21–65 years) recruited through the Human Immunology Core at the University of Pennsylvania. Whole blood (20–40 mL) was collected in EDTA tubes and processed immediately after collection to maintain neutrophil viability. Neutrophil isolation was performed using the MACSxpress Whole Blood Neutrophil Isolation Kit protocol (130-104-434, Miltenyi Biotec) following the manufacturer’s protocol. For each 8 mL whole blood, an isolation mix was prepared by combining 2 mL reconstituted antibody cocktail with 2 mL Buffer B. The isolation mix was added to anticoagulated whole blood in a 15 mL tube at a ratio of 1:2, gently inverted 3 times, and incubated for 15 minutes at room temperature on a tube rotator. The tube was then placed in a magnetic separator for 15 minutes. The supernatant containing untouched neutrophils was carefully collected. To assess neutrophil purity, cytospin preparations were made using 2 × 10^5^ cells centrifuged at 400 *g* for 5 minutes onto glass slides. Slides were fixed in 4% formaldehyde for 10 minutes and immunostained with anti-NE antibody (AB68672, Abcam, 1:200) followed by the appropriate secondary antibody and DAPI counterstaining. Neutrophil purity was consistently greater than 95% as determined by counting NE^+^ cells. Isolated neutrophils were used immediately for experiments.

#### Neutrophil coculture.

All coculture experiments utilized neutrophils from healthy donors. For coculture experiments, freshly isolated healthy donor neutrophils were cultured either alone or with primary human keratinocytes or fibroblasts at a 1:2 ratio in keratinocyte culture medium in 24-well plates. Primary human keratinocytes were obtained from neonatal foreskins (IRB exempt from the SBDRC Core A) and used between passages 2–5. Cell viability was assessed at 24, 48, 72, 96, 120, 144, and 168 hours using trypan blue and a LIVE/DEAD staining kit (L3224, Thermo Fisher Scientific). For neutrophil and T cell coculture experiments, healthy donor neutrophils were cultured with autologous purified T cells at a 1:1 ratio in complete RPMI medium supplemented with 10% FBS for the assay-dependent time period. Cocultures were maintained for 24–72 hours depending on the specific assay. All experiments were performed in triplicate using neutrophils from at least 3 independent healthy donors.

#### RNA isolation and quantitative real-time PCR.

Total RNA was isolated using TRIzol reagent (15596026, Thermo Fisher Scientific) and the RNeasy Mini Kit (74106, Qiagen) according to the manufacturer’s protocols. RNA concentration and purity were measured using the NanoDrop 1000 spectrophotometer (Thermo Fisher Scientific). cDNA synthesis was performed using the QuantiTect Reverse Transcription Kit (Qiagen) according to the manufacturer’s instructions. Quantitative real-time PCR (qRT-PCR) was performed on an ABI ViiA7 Real-Time PCR detection system (Applied Biosystems) using TaqMan Gene Expression Master Mix or SYBR Green Master Mix.

#### Drug and antibody treatment.

Neutrophil-keratinocyte cocultures were established for 48 hours before adding the following drugs at varying concentrations: prednisone (10 μM, 100 μM, 500 μM, 5 mM), dapsone (0.32, 3.26, 32.6, 326 μg/mL), anakinra (0.1, 1, 10, 100 ng/mL), and adalimumab (0.1, 1, 10, 100 μg/mL). After 48 hours of drug exposure (total 96-hour coculture), cell viability was assessed. Drug concentrations were chosen to reflect the therapeutic levels observed in clinical settings for Sweet syndrome treatment. Neutrophil survival was compared with vehicle-treated controls, with all conditions performed in triplicate.

For antibody neutralization targeting the SAA1/FPR2 axis, neutrophil-keratinocyte cocultures were treated from 0 hours with (a) no antibody (control), (b) isotype IgG (4 μg/mL), (c) anti-FPR2 (4 μg/mL, NLS 1878, Novus), (d) anti-SAA1 (4 μg/mL, 16721-1-AP, Proteintech), or (e) both antibodies (4 μg/mL each). To maintain neutralizing activity throughout the experiment, antibodies were replenished at 24 and 48 hours. At 72 hours, cells were harvested, and neutrophil viability was assessed.

#### Recombinant SAA1 treatment experiments.

Freshly isolated neutrophils from healthy donors were treated with recombinant human SAA1 (SAA1-257H, Creative Biomart). Neutrophils (5 × 10^5^ cells/well) were seeded in 24-well plates in keratinocyte medium supplemented with recombinant human SAA1 at concentrations of 5 μg/mL, twice a day. Cells were incubated at 37°C in 5% CO_2_ for up to 72 hours for survival assays or 24 hours for gene expression analysis. For survival assays, cell viability was assessed at 72 hours using trypan blue.

#### ELISA.

Neutrophil and keratinocyte coculture supernatants were collected and stored at –80°C. CXCL10, CXCL11, and SAA1 levels were assessed using the Human I-TAC/CXCL10 (RAB0119-1KT, MilliporeSigma), Human I-TAC/CXCL11 (RAB0121-1KT, MilliporeSigma), or the Human Serum Amyloid A1 DuoSet ELISA (DY3019-05, Biotechne) kits, respectively, following the manufacturers’ instructions. The ELISA was read at 450 nm. Between each step, the plate was washed at least 5 times with 1× PBS containing 0.05% Tween-20.

#### Histology and immunofluorescence staining.

Standard histology and immunostaining protocols were performed, and investigators were blinded to tissue origin during histologic staining (24). The Skin Biology and Disease Resource-Based Core (SBDRC) at the Department of Dermatology, University of Pennsylvania processed the slides and performed the H&E staining. H&E-stained sections were examined by a board-certified dermatopathologist under bright-field microscopy. For immunofluorescence microscopy, the following antibodies were used: CD3 (MCA1477, Bio-Rad), CD8 (170306S, Cell Signaling Technology), CD20 (14-0202-82, Thermo Fisher Scientific), CD68 (14-0688-82, Thermo Fisher Scientific), FCN1 (PA5-51552, Thermo Fisher Scientific), S100A8 (33254S, Cell Signaling Technology), S100A9 (34425S, Cell Signaling Technology), MHC-II (68258S, Cell Signaling Technology), IgG (66362S, Cell Signaling Technology), NE (AB68672, Abcam), ELANE (LS-B4244, LS-Bio), FPR2 (NLS1878, Novus), SAA1 (16721-1-AP, Proteintech), and MPO (sc-52707, Santa Cruz Biotechnology). The histology slides were imaged using tiled imaging at ×10 magnification on a Leica DM6B/DMC2900 imaging system (Leica Microsystems).

#### Cell migration assay.

T cell migration was assessed using a Transwell migration system. Purified CD3^+^ T cells were isolated from healthy donors and resuspended at 1 × 10^6^ cells/mL in keratinocyte medium. CM were collected from 72-hour cultures of keratinocytes alone or neutrophil-keratinocyte cocultures (1:2 ratio). Migration assays were performed in 24-well plates with 6.5 mm Transwell inserts (5.0 μm pore size, Corning). CM (600 μL) were added to the lower chamber, and 1 × 10^5^ T cells in 100 μL serum-free medium were added to the upper chamber. Plates were incubated for 2 hours at 37°C in 5% CO_2_. Migrated cells in the lower chamber were collected and counted using trypan blue staining. The percentage of migrated cells was calculated as follows: (number of cells in lower chamber/total input cells) × 100.

#### NETosis detection.

NET formation was assessed using immunofluorescence microscopy. Neutrophils, either freshly isolated or cocultured with keratocytes for 48 hours, were seeded at a density of 1 × 10^5^ cells on poly-L-Lysine-coated glass coverslips in 24-well plates and stimulated with 75 µg/mL lipopolysaccharide (LPS) (Cell Signaling Technology, 14011S) and cultured overnight at 37°C. Cells were fixed with 4% paraformaldehyde for 15 minutes, permeabilized with 0.1% Triton X-100 for 10 minutes, and blocked with 5% BSA for 1 hour at room temperature. Cells were incubated with anti–HLA-DR antibody (68258S, Cell Signaling Technology, 1:200) overnight at 4°C, followed by the appropriate fluorescent secondary antibody for 1 hour at room temperature. Cells were then stained with both DAPI (1:10,000) and SYTOX Green (1:5,000, Thermo Fisher Scientific) for 15 minutes. NETs were identified by extracellular DNA structures positive for both DAPI and SYTOX Green, appearing as diffuse, web-like structures extending beyond the original cell boundary. APC-like neutrophils were identified as cells with intact nuclei (DAPI^+^ SYTOX Green^–^) that expressed HLA-DR. Cells were categorized as: (a) normal neutrophils (intact nuclei, HLA-DR^–^); (b) APC-like neutrophils (intact nuclei, HLA-DR^+^); or (c) NET-forming neutrophils (extracellular DNA spread, DAPI^+^ and SYTOX Green^+^). Images were acquired using a Leica DM6B fluorescence microscope at ×20 magnification, capturing at least 10 random fields per condition. The percentages of NET-forming cells and HLA-DR^+^ cells were quantified separately.

### Computational and statistical analysis

#### scRNA-Seq data analysis.

The scRNA-Seq data were mapped to the GRCh38 reference genome to generate gene count and cell barcode matrices using the “cellranger count” function from the cellranger pipeline (version 5.0.1, 10X Genomics). All downstream analysis steps were performed using the R package in Seurat (25) (version 4.3.0, https://github.com/satijalab/Seurat) unless otherwise noted. In brief, the Seurat functions “Read10X” and “CreateSeuratObject” were used to import and create a merged Seurat object from all filtered feature barcode matrices generated by the cellranger pipeline. Cells with fewer than 250 genes, less than 500 unique molecular identifiers (UMI), less than 0.80 log_10_ genes per UMI, and more than 20% mitochondrial reads were excluded from the merged Seurat object for further analysis. Genes that were detected in fewer than 10 cells were also discarded. DoubletFinder was used to identify potential cell doublets as a final quality control (26). To determine the effect of the cell cycle, each cell was given a cell-cycle phase score using the Seurat function “CellCycleScoring” (27). The individual datasets were then log normalized and scaled by linear regression against the number of reads. The FindVariableFeatures function followed by the “SelectIntegrationFeatures” function (“nfeatures” = 3,000) were used to identify variable genes from each individual Seurat object. For cross-tissue data integration and batch correction, “FindIntegrationAnchors” and “IntegrateData” were applied to the individual sample Seurat object. Following sample integration, dimensionality reduction was performed using the RunPCA and RunUMAP function generated uniform manifold approximation and projection (UMAP) plots. Next, Louvain clustering was performed with the “FindClusters” function using the first 40 PCs and at a resolution of 1.4. The ElbowPlot function in Seurat, visual inspection of DimHeatmap plots at different dimensions, and R package clustree were used to choose an optimum number of dimensions and resolution.

#### Cell type annotation.

We used 3 complementary approaches to annotate the identities of different cell clusters. First, we checked the expression of lineage-specific marker genes identified from previously published scRNA-Seq studies in our query cluster marker genes list and in differentially expressed genes of the query cluster. Second, we applied an unbiased cell type recognition method named deCS (R package) (28), which leverages mapping of the top 100 genes from the query cluster to the reference transcriptomic datasets of known cell types such as BlueprintEncode (29), MonoccoImmune reference (30), and Database of Immune Cell Expression (DICE) data (31). We first applied deCS to determine if the predicted annotations were consistent with our findings and then assigned the identity to the cluster. And third, we described our methods to confirm skin and blood neutrophil identities in the primary text. The sample statistics and marker gene dot plots were made using dittoSeq, version 1.4.1. The *t*-distributed stochastic neighbor embedding (*t*-SNE) was applied to visualize the single-cell transcriptional profile in 2D space based on the SNN graph described above (32). Other bar plots, box plots, violin plots, and heatmaps were generated by customized R code through ggplot2, version 3.2.1 (R package) (33).

#### Ligand-receptor interaction analysis.

We used R package CellChat (1.5.0) to study the ligand-receptor interaction networks between different immune cell subclusters. We performed the ligand-receptor interaction analysis on the immune subcluster from the scRNA-Seq dataset. The analysis was performed on the paracrine signaling network. For our analysis, we considered ligand-receptor interactions that were expressed in at least 10 cells. The CellChat algorithm calculates an aggregated ligand-receptor interaction score on the basis of a method called “trimean.” The CellChat algorithm has the added advantage of comparing 2 or more single-cell datasets and gives a comparative score for the given cell types. These scores represent the probability of interaction among the ligand-receptor pairs. The probability was then visualized using functions such as netAnalysis_signalingRole_scatter, which visualizes the major sender and receiver across all cell types, and netAnalysis_signalingChanges_scatter, which identifies the major signaling networks acting within a given cell type.

#### Functional enrichment analysis.

For gene ontology (GO) analysis comparing skin versus blood neutrophils (Figure 2E), differential gene expression was performed using the Seurat package (version 4.3.0) FindMarkers function with the following parameters: thresh.use = 0.25, min.pct = 0.1, and logfc.threshold = 0.25. Genes were considered differentially expressed with an adjusted *P* value of less than 0.05 and |log_2_ fold change| >0.25. The top significantly upregulated genes in skin neutrophils were selected on the basis of an adjusted *P* value and submitted to Enrichr web tool (https://maayanlab.cloud/Enrichr/) for functional enrichment analysis. GO molecular function and Kyoto Encyclopedia of Genes and Genomes (KEGG) pathway analyses were performed through Enrichr. Enrichment results were downloaded and ranked by combined score, which integrates the *P* value and *z* score to assess both significance and effect size. The top pathways from each category were selected on the basis of the combined score. Results were visualized using GraphPad Prism 9.0 (GraphPad Software). Complete differential expression results are provided in Supplemental Table 4, with detailed pathway and GO enrichment data presented in Supplemental Table 5. All data in the plots are defined as mean ± SEM. 

### Statistics

The data presented in this work combined all experiments and, unless otherwise noted, all experiments were repeated 2–3 times independently. Experiments were not randomized, and investigators were not blinded to allocation during the experiments and outcome assessment, unless otherwise noted in the text. Comparisons between 2 groups were carried out using 2-tailed, unpaired Student’s *t* test and between multiple groups were carried out using 1-way ANOVA. In all tests, a *P* value of less than 0.05 was considered significant. When appropriate, specific *P* values are provided in Figure legends.

### Study approval

Human patients diagnosed with Sweet syndrome were recruited for this study at the Perelman Center for Advanced Medicine, University of Pennsylvania. Written informed consent was obtained before participation in the study under a protocol approved by the IRB of the University of Pennsylvania School of Medicine (IRB no. 832147).

### Data availability

Underlying data are provided in the Supporting Data Values file. All sequencing data have been deposited in the Gene Expression Omnibus (GEO) database and are publicly available (GEO GSE291004; https://www.ncbi.nlm.nih.gov/geo/query/acc.cgi?acc=GSE291004). This article does not report the original code. Any additional information required to reanalyze the data reported herein is available from the corresponding author upon request.

## Author contributions

MR, LZ, and RGM collected the clinical samples. JH, SS, and THL conceptualized the experiments, designed the study methodology, and performed experiments. SS performed bioinformatics analysis. THL wrote the manuscript. MR, LZ, OA, SS, and JH reviewed and edited the manuscript. For the 2 co–first authors, JH contributed more panels to the manuscript figures than SS. However, their contributions to the overall conclusions on the work were considered equal. ERR performed Microscopy data analysis.

## Supplementary Material

Supplemental data

Supplemental table 1

Supplemental table 2

Supplemental table 3

Supplemental table 4

Supplemental table 5

Supporting data values

## Version 1 08/19/2025

In-Press Preview

## Version 2 10/15/2025

Electronic publication

## References

[1] Cohen PR (2007). Sweet’s syndrome--a comprehensive review of an acute febrile neutrophilic dermatosis. Orphanet J Rare Dis.

[2] Nelson CA (2018). Neutrophilic dermatoses: Pathogenesis, Sweet syndrome, neutrophilic eccrine hidradenitis, and Behçet disease. J Am Acad Dermatol.

[3] Bhattacharya S (2022). Identification of a neutrophil-specific PIK3R1 mutation facilitates targeted treatment in a patient with Sweet syndrome. J Clin Invest.

[4] Heath MS, Ortega-Loayza AG (2019). Insights into the pathogenesis of sweet’s syndrome. Front Immunol.

[5] Joshi TP (2022). New practical aspects of sweet syndrome. Am J Clin Dermatol.

[6] Pang Z (2025). Spesolimab response in a patient with steroid-resistant sweet syndrome. JAMA Dermatol.

[7] Koenderman L (2022). Human neutrophil kinetics: a call to revisit old evidence. Trends Immunol.

[8] Moffat A, Gwyer Findlay E (2024). Evidence for antigen presentation by human neutrophils. Blood.

[9] Gosselin EJ (1993). Induction of MHC class II on human polymorphonuclear neutrophils by granulocyte/macrophage colony-stimulating factor, IFN-gamma, and IL-3. J Immunol.

[10] Grieshaber-Bouyer R (2022). Ageing and interferon gamma response drive the phenotype of neutrophils in the inflamed joint. Ann Rheum Dis.

[11] Vono M (2017). Neutrophils acquire the capacity for antigen presentation to memory CD4^+^ T cells in vitro and ex vivo. Blood.

[12] Sharma S (2016). A subset of neutrophils expressing markers of antigen-presenting cells in human visceral leishmaniasis. J Infect Dis.

[13] Jin H (2023). Antigen-presenting aged neutrophils induce CD4+ T cells to exacerbate inflammation in sepsis. J Clin Invest.

[14] Beauvillain C (2007). Neutrophils efficiently cross-prime naive T cells in vivo. Blood.

[15] Polak D, Bohle B (2022). Neutrophils-typical atypical antigen presenting cells?. Immunol Lett.

[16] Tabula Sapiens C (2022). The Tabula Sapiens: A multiple-organ, single-cell transcriptomic atlas of humans. Science.

[17] Wong A (2024). Ischemia-reperfusion responses in human lung transplants at the single-cell resolution. Am J Transplant.

[18] Hao Y (2021). Integrated analysis of multimodal single-cell data. Cell.

[19] Montaldo E (2022). Cellular and transcriptional dynamics of human neutrophils at steady state and upon stress. Nat Immunol.

[20] Calabrese L (2024). Molecular characteristics of sweet syndrome: a systematic review. Exp Dermatol.

[21] Chieosilapatham P (2024). Comparative immunohistochemical analysis of inflammatory cytokines in distinct subtypes of Sweet syndrome. Front Immunol.

[22] Kersh AE (2024). CXCL9, CXCL10, and CCL19 synergistically recruit T lymphocytes to skin in lichen planus. JCI Insight.

[23] Sati S (2024). Recruitment of CXCR4+ type 1 innate lymphoid cells distinguishes sarcoidosis from other skin granulomatous diseases. J Clin Invest.

[24] Huang J (2024). Granulocyte colony stimulating factor promotes scarless tissue regeneration. Cell Rep.

[25] Stuart T (2019). Comprehensive integration of single-cell data. Cell.

[26] McGinnis CS (2019). DoubletFinder: doublet detection in single-cell RNA sequencing data using artificial nearest neighbors. Cell Syst.

[27] Tirosh I (2016). Single-cell RNA-seq supports a developmental hierarchy in human oligodendroglioma. Nature.

[28] Pei G (2022). deCS: a tool for systematic cell type annotations of single-cell RNA sequencing data among human tissues. Genomics Proteomics Bioinformatics.

[29] Stunnenberg HG (2016). The international human epigenome consortium: a blueprint for scientific collaboration and discovery. Cell.

[30] Monaco G (2019). RNA-Seq signatures normalized by mRNA abundance allow absolute deconvolution of human immune cell types. Cell Rep.

[31] Schmiedel BJ (2018). Impact of genetic polymorphisms on human immune cell gene expression. Cell.

[32] Becht E (2018). Dimensionality reduction for visualizing single-cell data using UMAP. Nat Biotechnol.

