# Supplementary material for: SAA1/FPR2 signaling between keratinocytes and neutrophils sustains chronic inflammation in Sweet syndrome
Source: J Clin Invest. 2025 Aug 19;135(20):e193566. doi: 10.1172/JCI193566 (PMC12520672; doi:10.1172/JCI193566)
Supplement: Supplemental data [file jci-135-193566-s062.pdf]

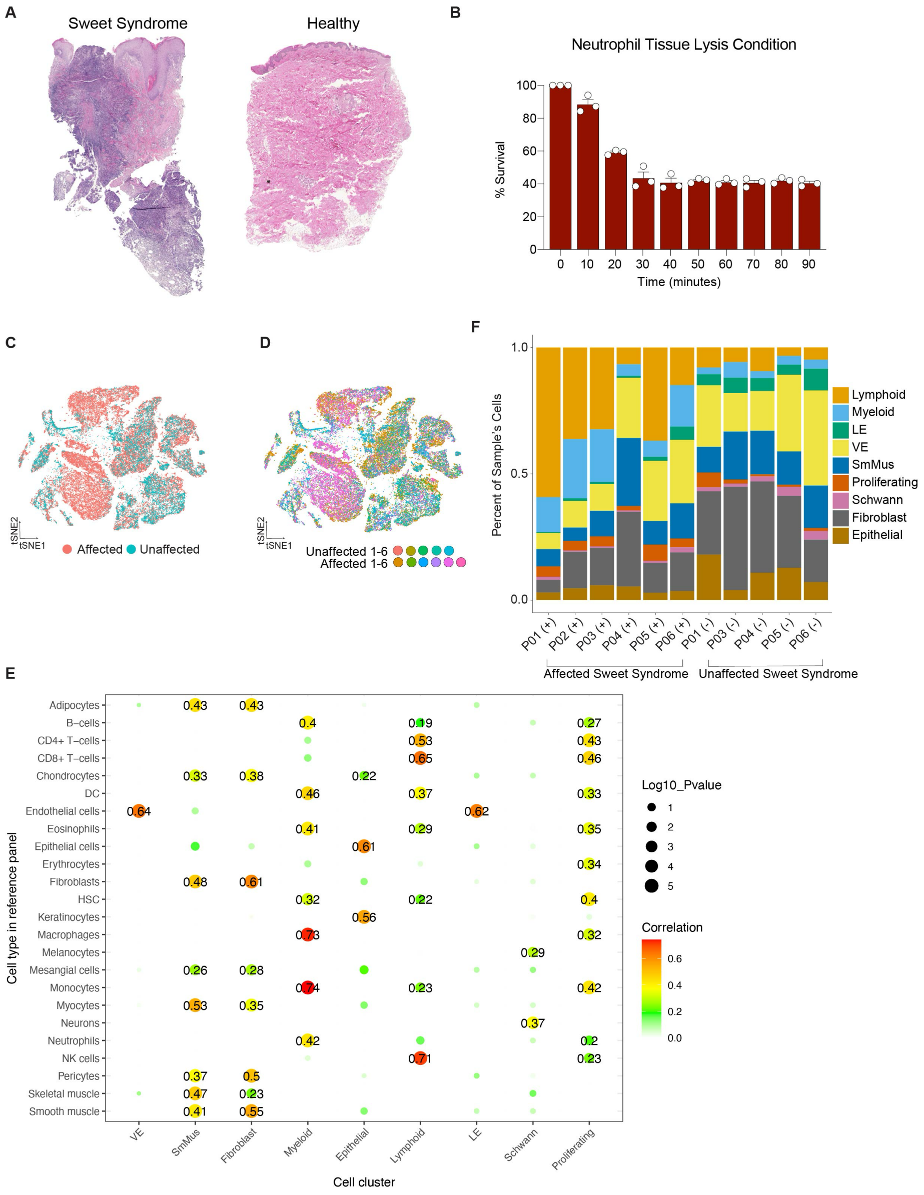

Supplemental Figure 1: Human Sweet syndrome samples. (**A**) H&E staining of affected and unaffected skin from Sweet syndrome patients. (**B**) Viability of blood neutrophils during the tissue dissociation protocol at different time points. Mean ± SEM from n=3 independent experiments. (**C**) tSNE plot demonstrating cell clusters overlap between affected and unaffected Sweet syndrome skin. (**D**) Cell contributions to tSNE plot from individual patient samples. (**E**) Cell type correlation analysis (deCS) mapping identified clusters to reference immune cell databases. Color intensity represents Pearson correlation coefficient; dot size indicates -log10 p-value. (**F**) Contributions of individual cell types in each patient sample.

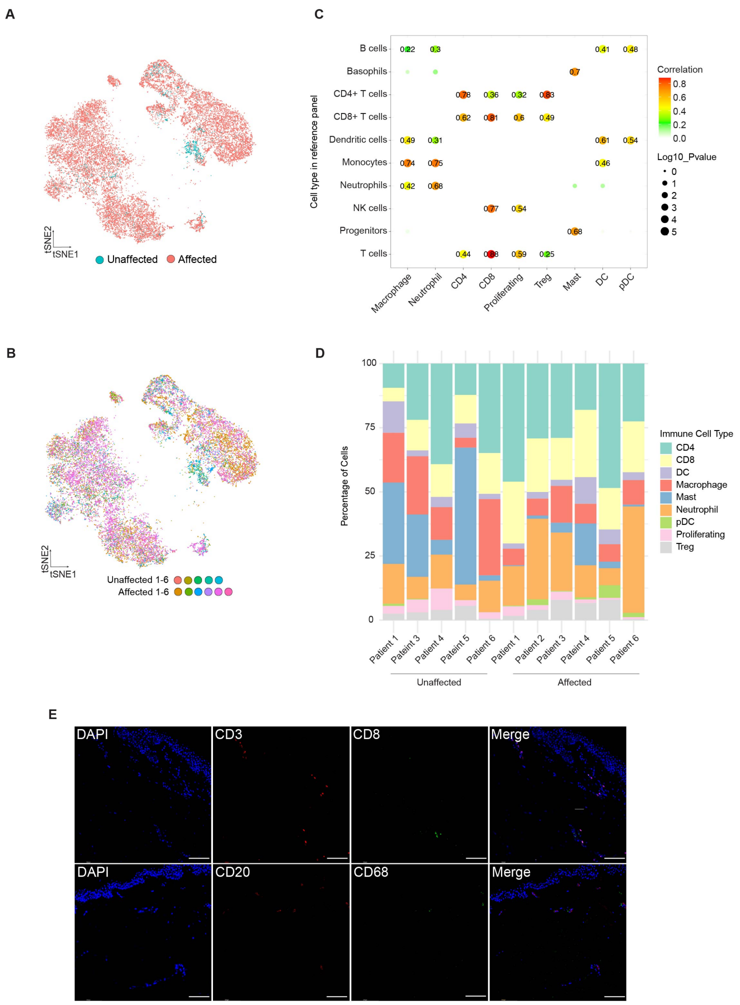

Supplemental Figure 2: Immune cell subclustering of Sweet syndrome skin

(A) tSNE plot of immune cell subcluster in unaffected and affected Sweet syndrome skin. **(B)** Contribution to tSNE plot by individual patients. **(C)** deCS correlation plot for cell type enrichment analysis of different cell clusters. Y-axis: main cell types identified from MonaccoImmune database. Color scale represents Pearson correlation coefficient, and dot sizes represent the -log10 transformed p value. (**D**) Contributions of individual immune cell types in each patient sample. (**E**) Representative immunofluorescence staining from 3 control human skin samples confirming populations of macrophages (CD68+), B cells (CD20+) and T cells (CD3+, CD8+) within normal human dermis. Scale bar 100µM.

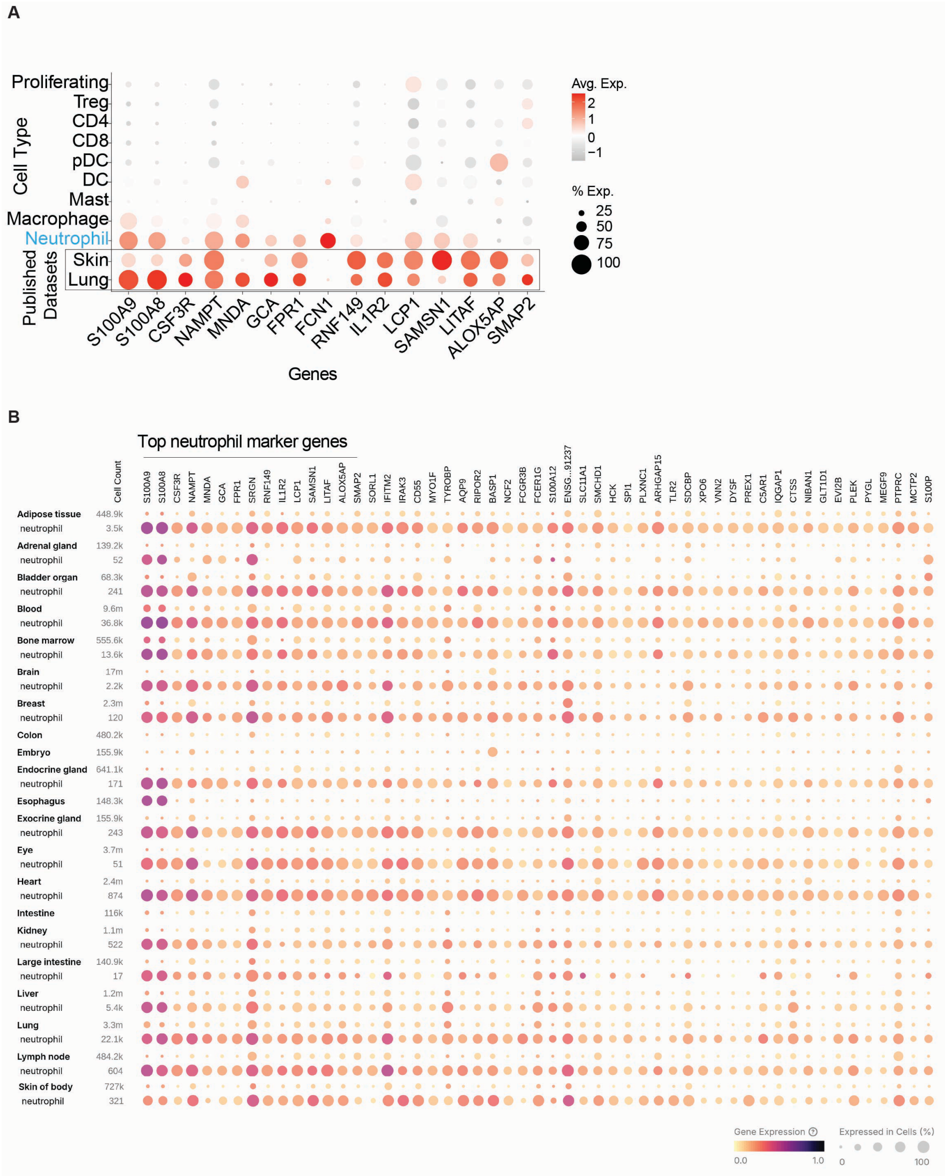

Supplemental Figure 3: Validation of neutrophil identity through marker gene expression. (**A**) Top 15 genes expressed in single-cell neutrophil cluster compared with other cell types and with published human neutrophil datasets. (**B**) Comprehensive dot plot of neutrophil marker gene expression across multiple human tissue sources from the Human Cell Atlas datasets.

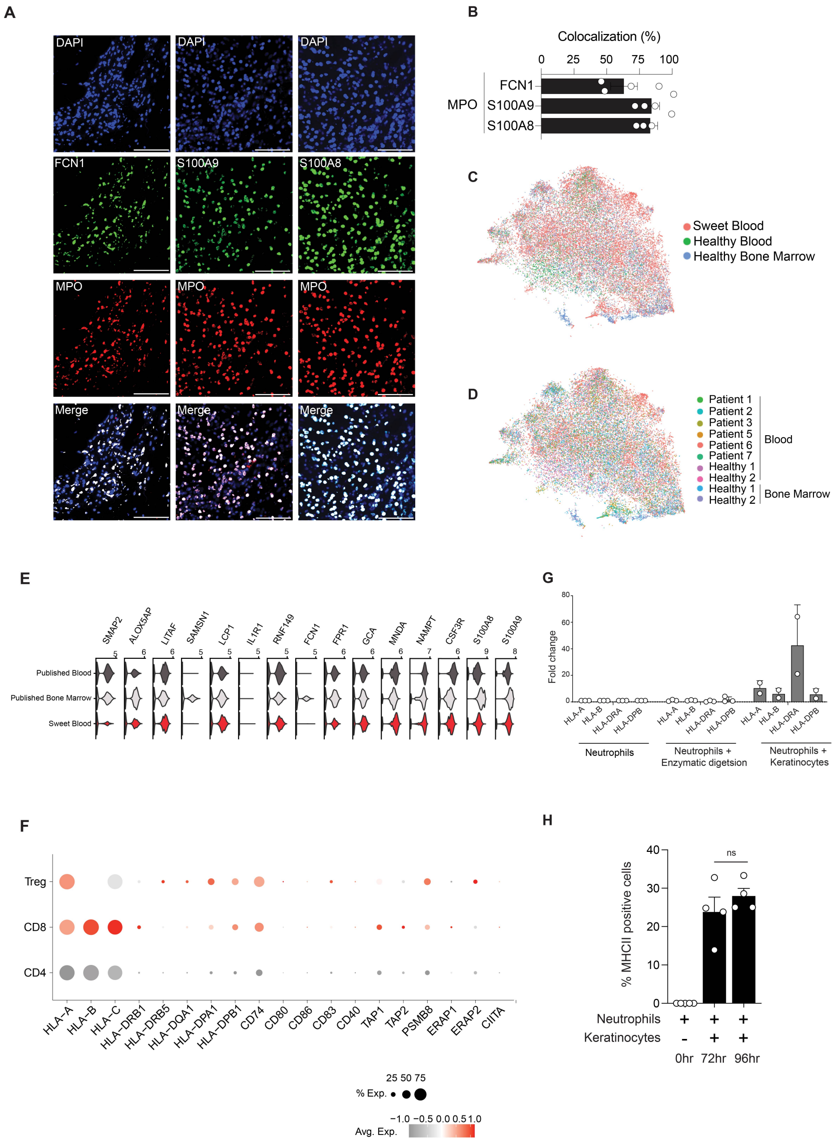

Supplemental Figure 4: Characterization of Sweet syndrome neutrophils.
(**A**) Immunofluorescence staining of Sweet syndrome tissue demonstrating co-expression of neutrophil-specific marker genes (FCN1, S100A8, S100A9) and myeloperoxidase (MPO). (**B**) Quantification of Immunostaining. n=4. **(C)** tSNE plot neutrophil subcluster showing overlap with published purified neutrophil from blood and bone marrow. (**D**) Contributions by each patient sample. (**E**) Shared expression of most of the top 15 neutrophil-specific genes identified in our skin dataset within blood neutrophils from different sources. (**F**) Dot plot showing MHC genes in different T cell subsets within Sweet syndrome blood. Dot size reflects percent cells expressing the gene, and color illustrates level of gene expression. (**G**) qPCR analysis of MHC gene expression in healthy neutrophils following enzymatic digestion or keratinocyte co-culture (positive control). (**H**) Immunofluorescence quantification of MHC-II positive neutrophils after 72 or 96 hours of culture with or without keratinocytes. One-way ANOVA with individual comparisons. ns, not significant. Mean ± SEM, n=3.

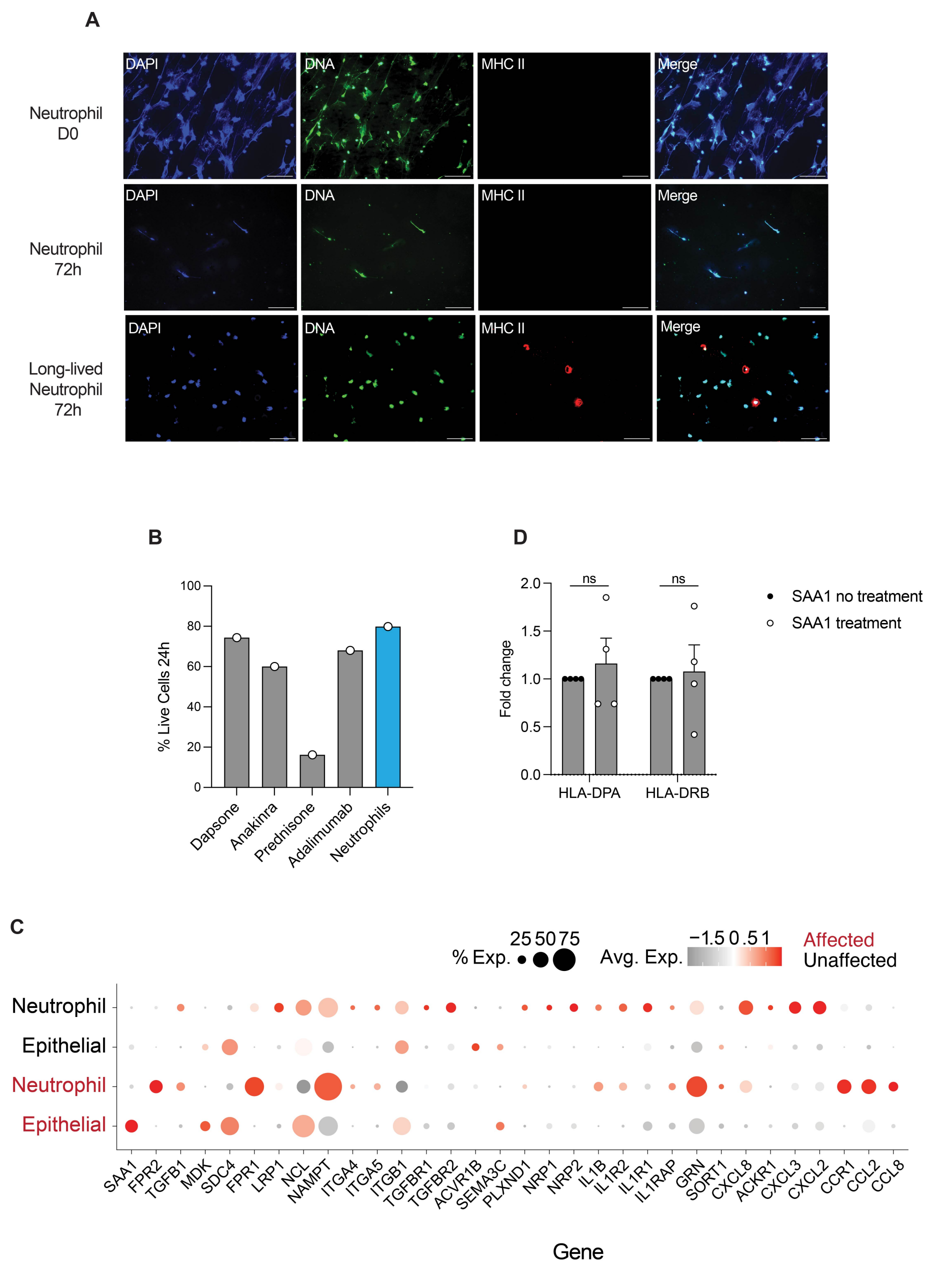

Supplemental Figure 5: Long-lived neutrophils exhibit reduced spontaneous NETosis, *SAA1* and *FPR2* transcripts are induced in Sweet syndrome skin.

(**A**) Immunostaining depicting decreased spontaneous NETosis in long-lived neutrophils (DAPI, blue, DNA, green, MHC, red). Scale bars 100µM. (**B**) Effect of Sweet syndrome therapeutics on healthy neutrophil survival at 24 hours. (**C**) Compared to unaffected control, dot plot demonstrating induction of *SAA1* and *FPR2* transcript in affected skin keratinocytes and neutrophils, respectively. Dot size reflects percent cells expressing the gene, and color illustrates level of gene expression. (**D**) qPCR analysis assessing MHC class II gene expression (*HLA-DPA, HLA-DRB*) in neutrophils at 24 hours after treatment with and without SAA1 treatment. Two-tailed Student's T-test. ns, not significant. Mean ± SEM, n=4.
